# The psychological characteristics behind talented soccer players: a systematic review with meta-analysis

**DOI:** 10.3389/fpsyg.2026.1821294

**Published:** 2026-05-06

**Authors:** Filipe Casanova, José Afonso, Alberto Pompeo, José Vilaça-Alves, Nuno Domingos Garrido, Everton Luis Rodrigues Cirillo, Rodrigo Ramirez-Campillo, Pedro Silva, André Barreiros

**Affiliations:** 1 Universidade Lusófona do Porto, Porto, Portugal; 2 Centro de Investigação em Desporto, Educação Física, Exercício e Saúde (CIDEFES), Porto, Portugal; 3 Núcleo de Estudos e Investigação em Futebol (NEIF), Porto, Portugal; 4 Faculty of Sport, University of Porto, Porto, Portugal; 5 Centre for Research, Education, Innovation and Intervention in Sport (CIFI2D), University of Porto, Porto, Portugal; 6 Universidade de Trás-os-Montes e Alto Douro, Vila Real, Portugal; 7 Research Center in Sports Sciences, Health Sciences & Human Development, CIDESD, Vila Real, Portugal; 8 Research Group in Strength Training and Fitness Activities (GEETFAA), Vila Real, Portugal; 9 Universidade Estadual de Londrina, Londrina, Brazil; 10 Department of Physical Activity Sciences, Universidad de Los Lagos, Osorno, Chile; 11 Universidad de Tarapacá, Arica, Chile; 12 Exercise and Rehabilitation Sciences Institute, Faculty of Rehabilitation Sciences, Universidad Andres Bello, Santiago, Chile; 13 Instituto Superior Manuel Teixeira Gomes, Portimão, Portugal; 14 Sport, Physical Activity and Health Research & Innovation Center, Viana do Castelo, Portugal

**Keywords:** football, psychology, recruitment, selection, sports

## Abstract

Soccer performance depends on a long-term process, with the psychological characteristics of young talented players being influential. This meta-analytic review synthesizes research that examined the psychological factors associated with talented soccer players, providing guidance for future studies. This study asked whether psychological factors are associated with future talent-like performance in young football players. We identified 4,015 records by searching PubMed, Scopus, and Web of Science. Of the 1,511 records screened, only 12 studies were included, as they were designed to be longitudinal and covered 3,748 players across recreational to elite levels. Risk of bias was assessed using the Risk of Bias Assessment Tool for Nonrandomized Studies, and certainty of evidence was judged with the GRADE framework. The findings suggest that greater values of achievement motive correlated positively with future talent-like performance; however, the pooled effect size was merely trivial (*g* = 0.198, 95% CI: 0.003–0.378) and heterogeneity was large (*I*^2^ = 78.5%), likely reflecting differences in measurement tools, sample characteristics, and performance indicators. No other psychological construct could be meta-analysed due to insufficient and heterogeneous data, and overall certainty of evidence for all outcomes was rated very low. Other psychological characteristics such as mental toughness, grit, and resilience may play a functional role in talent development, but their isolated prognostic value for future performance appears small, inconsistent, and highly sensitive to methodological choices. In the future psychological factors should therefore not be used as standalone criteria for talent selection or deselection, but rather integrated within multidimensional, longitudinal assessment frameworks rather than solely cross-sectional designs, that combine technical, tactical, physical, and contextual information.

Systematic review registration: 10.17605/OSF.IO/J4EPT

## Introduction

Forty years have passed since Bloom’s seminal publication about talent development (Bloom, 1985), a turning point in the understanding of the concept of talent. Talent is a development process that emerges through the interaction of different variables and according to a dynamic interplay between genetic and environmental factors (Phillips et al., 2010; Simonton, 1999). Over time, an impressive number of investigations have been conducted in diverse contexts, with different methodological designs and studying multiple variables (Ivarsson et al., 2020; Murr et al., 2018). Sport has probably been one of the contexts where the most investment has been made to study talent development and to use that knowledge in applied settings. Soccer clubs and academies are among the settings where more resources have been dedicated to this process.

As soccer performance depends on a long-term process of training different components (i.e., technical, tactical, physical and psychological), close attention has been given to the process of optimizing training environments with a particular emphasis on the psychological factors (Henriksen et al., 2020; Martindale and Mortimer, 2011; Xia et al., 2024). Understanding how these factors develop and how they may be managed by soccer players in training and competition is an essential question. In recent years, several studies reviewed the literature around this topic (Freitas et al., 2013; Gledhill et al., 2017; Haugaasen and Jordet, 2012; Ivarsson et al., 2020; Murr et al., 2018).

To provide a comprehensive overview about the factors contributing to the development of soccer expertise, Haugaasen and Jordet (2012) used the Developmental Model of Sport Participation as framework (Côté, 1999; Côté et al., 2007) to analyze 115 studies. The study emphasized the importance of early and balanced engagement in soccer-specific activities, the value of structured and unstructured practice (e.g., deliberate practice and deliberate play), the need for individualized development pathways, and the role of holistic support environments in developing soccer expertise. The authors also reviewed some psychological factors such as motivation to participate or to dropout, as well as the psychosocial influences related to these intentions and outcomes. Notwithstanding, they underlined the lack of longitudinal and field studies that could capture the “functional interaction between technical, psychological and physical” factors (Haugaasen and Jordet, 2012, p. 196).

Relatedly, Freitas et al. (2013) focused their review on the methodological procedures to study psychological skills training (PST) in soccer. From the methodology applied and from the 28 studies reviewed (published between 1982 and 2012), the authors concluded that the use of PST in soccer has grown, particularly since 2004. Moreover, they found that most studies used both qualitative and quantitative approaches, although essentially focusing non-elite and U16 athletes. In another perspective, Gledhill et al. (2017) analyzed 48 studies (2004 to 2016) about the psychosocial factors underpinning talent development in soccer; self-regulation, resilience, commitment and discipline were the most important psychological factors to the soccer player development and multiple social agents around the athlete (i.e., parents, siblings, coaches and peers) were shown to play crucial roles of support (e.g., tangible, emotional and informational).

Murr et al. (2018) published a systematic review about the psychological “predictors” of talent in youth soccer. From the 16 studies included, the authors found that important levels of methodological heterogeneity which difficult comparisons across studies, psychomotor and personality-related factors received more attention than the perceptual-cognitive ones, and motivational characteristics (i.e., achievement goals, motivational orientation) have received more attention as prognostic personality-related variables. More recently, Ivarsson et al. (2020) reviewed the role of the psychological factors on future performance of soccer players, and the authors made important efforts to establish a broad understanding of future soccer performance, including selection to a specific team or higher playing level, contract extension, professional contract, superior performance as evaluated by objective measures from match-analyses or subjective rating from coaches and academy directors, or the psychological factors on soccer performance. The authors found that the number of investigations with prospective designs were very limited. They further observed small effect sizes of the psychological factors (e.g., task orientation, coping strategies) on future soccer performance.

In sum, up until 2020, the longitudinal research of the relationship between psychological factors and soccer talent remained largely underexplored. Therefore, we systematically reviewed and meta-analysed the association of psychological factors with talented players across their soccer career, to critically review the psychological characteristics used in the included studies, and to summarize the evidence for the current research question, providing guidance for future studies.

## Methods

This systematic review was pre-registered on the Open Science Framework (OSF) platform on 21/01/2025, 12 days before the searches were performed (project: osf.io/jv3my; registration doi: 10.17605/OSF.IO/J4EPT). This review was planned, conducted, and reported following the PRISMA 2020 guidelines (Page et al., 2021) (checklist in Supplementary File 1), and Cochrane’s guidelines (Higgins et al., 2019).

### Eligibility criteria

Original research published in peer-reviewed journals was included, without restrictions concerning publication date or language, and with no filters applied. Eligibility criteria followed the P.E.C.O.S. approach:

- Soccer players with no restrictions concerning sex, age or participation level assessed through the Participant Classification Framework (PCF) (McKay et al., 2022) Tier 1 – Recreationally Active, Tier 2 – Trained/Developmental, Tier 3 – Highly Trained/National Level, Tier 4 – Elite/International Level and Tier 5 – World Class, but excluding Tier 0 (Sedentary); futsal, indoor soccer, beach soccer, Paralympic soccer and other variations were not considered;
- Exposed to a deliberate process of talent identification, detection, selection, and/or development while belonging to a soccer team;
- Comparators included players not classified as talented within the studies;
- Outcomes had to include an assessment of discriminant psychological characteristics of the players (e.g., personality, motivation, resilience, focus);
- Longitudinal studies with at least two groups (e.g., players classified as talented versus non-talented, either at the beginning of the study, or later in the process).

### Information sources

Searches were conducted on 02/02/2025 in PubMed, Scopus, and Web of Science (core collection), with no filters applied. Manual searches were conducted within the reference lists of the included studies. Afterwards, snowballing citation tracking was implemented to detect further potentially relevant studies. Two external experts (Ph.D., published research on the topic) were consulted via e-mail to provide further suggestions of potentially relevant studies. These experts were given information about the inclusion criteria and a list of already included studies. A waiting period of 4 weeks was allotted for the first response, with a reminder sent after the initial 2 weeks. In the case of a positive response, an additional four-week period was granted to complete the task. Finally, existing relevant errata or retractions was searched for.

### Search strategy

The general search strategy made use of free text terms and the Boolean operators AND/OR:

[Ti/Ab]: Soccer OR Football*.

*AND*

[Ti/Ab]: Talent*.

The full search strategies for each database are presented in Supplementary File 2.

### Selection process

Automated removal of duplicates was performed using EndNoteWeb (version 2024, Clarivate™, United States), but further manual removal of duplicates was required. Two reviewers (EC and AP), independently checked each record in two stages: (i) screening of titles and abstracts; (ii) full text analysis. In case of disagreements between the two authors, FC provided arbitrage until consensus was achieved.

### Data collection process

Two reviewers (EC and AP) independently collected data from reports. In case of disagreements between the two authors, FC provided arbitrage until consensus was achieved. Data was organized and coded using a specifically designed Microsoft® Excel (version 2024, USA) worksheet. Data on competitive level was re-coded by two authors (AP and FC) using the PCF (McKay et al., 2022). If any crucial data or contextual information was missing, the authors contacted via email and gave a four-week period for response (including a reminder after the first 2 weeks). If no response was received within 4 weeks, or the authors did not provide the requested data, and the missing information was deemed necessary based on the eligibility criteria, the study was excluded.

### Data items

*Participant-related information*: sample size, age, sex, competitive level according to PCF.

*Exposure-related information*: deliberate processes of identification, detection, selection, and/or development; also, details on years of practice.

*Outcome-related information*: psychological data (e.g., task and ego orientation, motivation, satisfaction, focus) across time (the studies were longitudinal).

*Other study-related information*: funding, competing interests.

### Study risk of bias assessment

Risk of bias was performed at outcome-level using Cochrane’s RoBANS (Park et al., 2011), with a worst-case scenario provided for the study level. Six domains were assessed: selection of participants, confounding variables, measurement of intervention (exposure), blinding of outcome assessment, incomplete outcome data, and selective outcome reporting. AP and JA independently assessed risk of bias. In case of disagreements between the two authors, FC provided arbitrage until consensus was achieved.

### Effect measures and synthesis methods

Although meta-analysis can be computed using two studies (Valentine et al., 2010; García-Hermoso et al., 2019; Skrede et al., 2019), sports science studies usually have small samples (Abt et al., 2020; Lohse et al., 2020). To avoid excessively small sample sizes, meta-analyses were conducted only if ≥3 studies provided data for a given outcome. The effect size (ES) estimate was based on correlation coefficients (*r*) for each outcome variable, alongside the corresponding standard errors or sample size. If data were reported in other statistical formats, conversion was performed using specialized software (Comprehensive Meta-Analysis Software, Version 4, Biostat, Englewood, NJ, United States), following international guidelines. The DerSimonian and Laird random-effects model was used to account for differences between studies that might affect the exposure effects (Deeks et al., 2024; Kontopantelis et al., 2013). The ES values (*r*) were presented with 95% confidence intervals (95% CIs), and the *true* ES in 95% of all comparable populations were calculated using the prediction interval (PI). Calculated ES was interpreted using the following scale: small (≤ 0.1), moderate (0.1–0.29) or large (≥ 0.30) (Cohen, 1988). The impact of study heterogeneity was assessed using the *I*^2^ statistics, with values of <25%, 25–75, and >75% representing low, moderate, and high levels of heterogeneity, respectively (Higgins and Thompson, 2002). All analyses were carried out using the Comprehensive Meta-Analysis Software (Version 4, Biostat, Englewood, NJ, United States). Statistical significance was set at *p* ≤ 0.05. Planned moderator analyses could not be implemented given the insufficient number of studies.

### Certainty assessment

Certainty or confidence in the body of evidence for each outcome was assessed using Grading of Recommendations Assessment, Development and Evaluation (GRADE) (Guyatt et al., 2011), considering its five dimensions (Schünemann et al., 2020a, 2020b). Particularly, evidence initiated as having high certainty and was downgraded based on the following criteria: (i) risk of bias in studies, with an average moderate risk being downgraded by one level, and high risk by two levels; (ii) indirectness, which was considered low risk due to the specificity of the populations, interventions, controls, and outcomes outlined in the eligibility criteria; (iii) the risk of publication bias was not considered due to concerns in its interpretation raised by Afonso et al. (2024); (iv) inconsistency, with a moderate impact of statistical heterogeneity (*I*^2^ ≥ 25%) resulting in a one-level downgrade and a high impact (*I*^2^ > 75%) resulting in a two-level downgrade; and (v) imprecision, with a one-level downgrade occurring if the comparison included less than 800 participants and/or if no clear direction of effects was noted (Guyatt et al., 2011). In cases where both imprecision criteria were observed, the level of certainty was downgraded by two levels. For outcomes where the number of comparison trials was insufficient for a meta-analytical approach, the evidence was judged to be of very low certainty.

## Results

### Study selection

**Figure 1:**
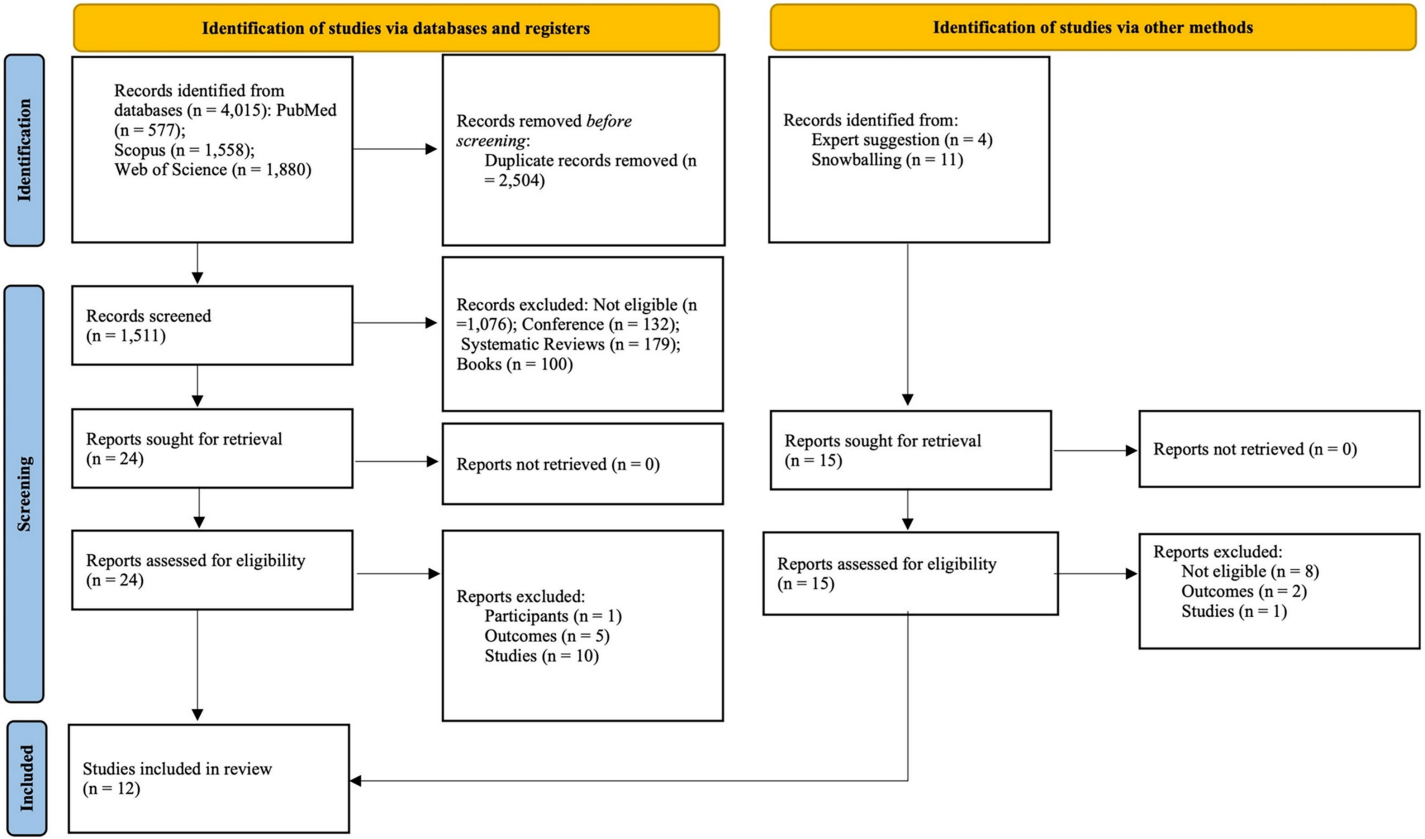
PRISMA 2020 flow diagram.

The search retrieved a total of 4,015 records (Figure 1): PubMed (577), Scopus (1,558), and Web of Science (1,880), of which 2,504 were duplicates. Of the 1,511 records screened, we excluded 132 conference proceedings, 100 books or book chapters, 179 reviews, and 1,076 were due to not fulfilling one or more P.E.C.O.S. criteria. Twenty-four studies were deemed eligible for full text analysis. Of these, one study was excluded since participants were from another sport (Berry et al., 2008), 5 studies were rejected because they did not assess psychological factors (Aquino et al., 2017; Bakhtiar et al., 2023; Barron et al., 2018; Bidaurrazaga-Letona et al., 2019; Elferink-Gemser et al., 2012) and 10 studies were cross-sectorial (Abarghoueinejad et al., 2021; Amar et al., 2023; Barraclough et al., 2022; Coelho e Silva et al., 2010; de Oliveira et al., 2016; Engan and Sæther, 2018; Mitchell et al., 2024; Ommundsen et al., 2005; Orosz and Mezo, 2015; Vidigal et al., 2022).

Therefore, 8 studies were included after the database searches (Figueiredo et al., 2009; Hendry et al., 2019; Forsman et al., 2016; Höner and Feichtinger, 2016; Huijgen et al., 2014; Rongen et al., 2020; Verner-Filion and Gaudreau, 2023; Zuber et al., 2022). The reference lists of these studies were then searched to retrieve potentially relevant titles that had not emerged in our initial searches; however, no additional studies were found. We also performed a snowballing citation tracking for the 8 studies included in Web of Science on March 2nd of 2025, and two external experts were also contacted. Of the 15 identified records, 8 did not fulfil the P.E.C.O.S. criteria. Of the 7 remaining records, one was cross-sectorial (Reilly et al., 2000), and two did not assess the psychological factor (Savelsbergh et al., 2020; Wilson et al., 2016). Four studies suggested by the experts were included (Van Yperen, 2009; Zuber and Conzelmann, 2013; Zuber et al., 2014; Zuber et al., 2016). Ultimately, 12 studies were included (Figueiredo et al., 2009; Hendry et al., 2019; Forsman et al., 2016; Höner and Feichtinger, 2016; Huijgen et al., 2014; Rongen et al., 2020; Van Yperen, 2009; Verner-Filion and Gaudreau, 2023; Zuber and Conzelmann, 2013; Zuber et al., 2014; Zuber et al., 2016; Zuber et al., 2022). Figure 1 synthesizes the search and selection process.

### Study characteristics

The samples varied from 40 (Rongen et al., 2020) to 2,677 players (Höner and Feichtinger, 2016), with a total of 3,748 players (99.17% male and 0.83% female) from T1 (Recreationally Active) to T4 (Elite/International Level) (McKay et al., 2022). The data items are presented in Table 1.

**Table 1:** Characteristics of the included studies.

Study	Aim	Participants (*n*; sex)	Age (years)	Competitive level (tier)	Instruments
Figueiredo et al. (2009)	To compare the baseline physical growth and biological maturity status, functional capacities, sport-specific skills, and goal orientation of youth soccer players who subsequently discontinued participation in the sport (drop-out), continued to participate at the same level (club) or moved to a higher playing standard (elite)	159 Male	11.0 to 14.9	T2	Task and Ego Orientation in Sport Questionnaire (TEOSQ)
Forsman et al. (2016)	To examine which technical, physiological, tactical and psychological characteristics at age 15 years contribute to successful soccer performance at age 19 years	114 Male	15.4 ± 0.3	T2 and T3	Psychological Skills Inventory for Sports (PSIS-R-5)
Hendry et al. (2019)	To determine if self-determined motivation (SDM) in elite men’s soccer changes over time and differs as a function of age, skill-grouping, and engagement in soccer play and practice	63 Male	12 to 17	T2 and T3	Behavioral Regulation in Sport Questionnaire (BRSQ2)
Höner and Feichtinger (2016)	To examine the relationship of talented soccer players’ psychological characteristics with current and future performance.	2,677 Male	11 and 12	T2 and T3	Achievement Motives Scale-Sport (AMS-S), Sport Orientation Questionnaire (SOQ), TEOSQ, Volitional Components in Sport questionnaire (VCS), Self-Efficacy in Soccer questionnaire (SES), Competition Anxiety Inventory Trait (CAI-T)
Huijgen et al. (2014)	To examine whether performance characteristics discriminated between selected and deselected players in talent development programmes	113 Male	12 to 18	T2 and T3	TEOSQ, Psychological Skills Inventory for Sports (PSIS-Youth)
Rongen et al. (2020)	To track psychosocial outcomes of academy involvement within male youth elite soccer players compared to age-matched soccer-active school pupils over 12 months.	40 Male	12 to 16	T1 and T2	Balanced Measure of Psychological Needs (BMPN), KIDSCREEN-27, Athletic Identity Measurement Scale (AIMS)
Van Yperen (2009)	To identify psychological factors that predict career success in professional adult soccer	65 Male	16.58 ± 1.40	T2 to T4	Self-report measure of goal commitment, The Ways of Coping Questionnaire
Verner-Filion and Gaudreau (2023)	To investigate the role of motivational factors as predictors of selection into competitive youth soccer teams, over and above the relative age effect	67 Male (*n* = 36) Female (*n* = 31)	11 to 13 (12.29 ± 0.65)	T2	Basic Need Satisfaction Scale, Sport Motivation Scale (SMS)
Zuber and Conzelmann (2013)	To determine which model best explains how hope for success and fear of failure To identify which are the aspects of the achievement motive, motor skills and abilities that affect performance	140 Male	12 to 15 12.26 ± 0.29	T2	AMS-S
Zuber et al. (2014)	To analyse the influence that motivational characteristics have on the development of performance, in a person-oriented way	97 Male	12 to 15 12.24 ± 0.29	T3	AMS-S, SOQ, SMS
Zuber et al. (2016)	To analyse the patterns formed by the constructs net hope, motor abilities, technical skills and biological maturity were examined, as well as the way in which these holistic patterns are related to subsequent sporting success	119 Male	12 to 15 12.27 to 14.33	T1 to T3	AMS-S
Zuber et al. (2022)	To examine whether motivational patterns of U14 soccer players were associated with successful soccer performance at the U19 age group	94 Male	13 to 19 13.31 ± 0.29	T2 to T4	AMS-S, SOQ, SMS

All the studies used questionnaires as the preferred instrument to assess and track the psychological characteristics of the soccer players. However, dissimilar questionnaires were used depending on the aims. The most commonly used questionnaires were AMS-S (Höner and Feichtinger, 2016; Zuber and Conzelmann, 2013; Zuber et al., 2014; Zuber et al., 2016; Zuber et al., 2022), TEOSQ (Figueiredo et al., 2009; Höner and Feichtinger, 2016; Huijgen et al., 2014), SOQ (Höner and Feichtinger, 2016; Zuber et al., 2014; Zuber et al., 2022), and SMS (Verner-Filion and Gaudreau, 2023; Zuber et al., 2014; Zuber et al., 2022).

### Risk of bias in studies

Table 2 presents the risk of bias analysis using Cochrane’s RoBANS (Park et al., 2011). Risk of bias was high across the board, with 10 of 12 studies (83.3%) having between 1 and 3 domains judged at high risk of bias, and the remaining two studies having some concerns. Regarding selective outcome reporting, no study had a pre-registered protocol, so the assessment was deemed unclear by default. Half of the studies had high risk of bias regarding incomplete outcome data, but this should likely be expected for most longitudinal studies. High risk of bias was prevalent for confounding, as many relevant variables (e.g., maturation level, training history) were not always appropriately monitored and could have affected the outcomes.

**Table 2:** Risk of bias (RoBANS) of the included studies.

Article	Selection of participants	Confounding variables	Measurement	Blinding outcome assessment	Incomplete outcome data	Selective outcome reporting
Figueiredo et al. (2009)	low	unclear	low	high	low	unclear
Forsman et al. (2016)	low	high	low	unclear	high	unclear
Hendry et al. (2019)	low	high	low	unclear	high	unclear
Höner and Feichtinger (2016)	low	unclear	low	unclear	low	unclear
Huijgen et al. (2014)	low	high	low	unclear	high	unclear
Rongen et al. (2020)	low	unclear	low	high	low	unclear
Van Yperen (2009)	low	high	low	unclear	high	unclear
Verner-Filion and Gaudreau (2023)	low	high	low	unclear	high	unclear
Zuber and Conzelmann (2013)	low	high	low	unclear	low	unclear
Zuber et al. (2014)	low	high	low	unclear	low	unclear
Zuber et al. (2016)	low	unclear	low	unclear	low	unclear
Zuber et al. (2022)	low	high	low	unclear	high	unclear

Colours represent risk of bias judgments: green (low risk), yellow (unclear risk), and red (high risk).

### Results of individual studies

The results of each study included in this research are presented in Table 3.

**Table 3:** Results of individual studies.

Study	Type of study	Duration and tracking periods (TP)	Psychological characteristics (and categories)	Main findings
Figueiredo et al. (2009)	Longitudinal	2 years (TP1–2003; TP2–2005)	Task and ego orientation	Task and ego orientation were apparently not related to the process of dropping out, persisting at the same competitive standard or moving up to a higher level in this sample of youth soccer players.
Forsman et al. (2016)	Longitudinal	4 years (TP1–2010; TP2–2014)	Self-determination (SDM; Self-motivation Index – SMI), confidence, concentration, mental preparation	The motivational skills were associated with the future performance level of youth soccer players.
Hendry et al. (2019)	Longitudinal	3 years (TP1–2011; TP2–2014)	SDM (SMI, autonomous and controlled motivation)	Elite youth players were generally more autonomously motivated than the non-elite athletes. Differences in SDM as a function of age and skill point toward the dynamic nature of these motivations over time, likely a result of proximity to external rewards. The absence of any relations between SDM and soccer play did not support a key prediction related to the Developmental Model of Sport Participation.
Höner and Feichtinger (2016)	Longitudinal	4 years (TP1–2010; TP2–2014)	Achievement motives (hope for success and fear of failure), achievement orientations (competition, win and goal orientation), task and ego orientation, volitional components (self-optimization, self-impediment, lack of initiation, and loss of focus), competition anxiety (somatic anxiety, worry, and concentration disruption)	Both components of the achievement motives assessed by the AMS-S were associated with future performance level, in which particularly hope for success was identified as prognostically relevant. The two goal orientations (i.e., task and ego orientation) showed no associations with current performance. However, task orientation was a significant predictor of future success. In comparison to the TEOSQ, the SOQ seems to be more appropriate for assessing motivational orientations in the context of sport talent research. Competition and goal orientation of the SOQ demonstrated relevant associations with current and future performance. Self-optimization was more important than all three volitional deficits.
Huijgen et al. (2014)	Longitudinal	<1 year	Task and ego orientation, motivation, self-confidence, anxiety control, mental preparation, team emphasis, concentration	The psychological characteristics did not differentiate selected from deselected players in.
Rongen et al. (2020)	Longitudinal	1 year (TP1 – October; TP2 – February; TP3 – May; TP4 – September)	Satisfaction, thwarting (dissatisfaction), emotional well-being, athletic identity (exclusivity, negative affectivity, social identity)	Academy players reported consistent significantly higher total athletic identity and exclusivity of identity. Findings suggest that many concerns around negative psychosocial impacts of soccer academy involvement did not materialise in this context. However, heightened athletic identities remained a concern.
Van Yperen (2009)	Longitudinal	15 years	Goal importance and goal commitment (goal commitment, exhaustion), coping (problem-focused coping, emotion-focused coping)	The psychological factors that predicted career success while statistically controlling for initial performance level and demographic variables were goal commitment, and engagement in problem-focused coping behaviours.
Verner-Filion and Gaudreau (2023)	Longitudinal	6 months (TP1 – October; TP 2 – April)	Satisfaction and SDM (autonomous, controlled motivation)	Experiencing high levels of need satisfaction is conducive to autonomous sport motivation which, in turn, was positively related to the likelihood of being selected in a competitive youth team.
Zuber and Conzelmann (2013)	Longitudinal	7 months	Achievement motives (hope for success, fear of failure)	The development of athletic performance in soccer depends on multiple factors, and in particular hope for success was worth watching in the medium term as a predictor of talent.
Zuber et al. (2014)	Longitudinal	1 year (TP – 2011; TP2 – 2012)	Achievement motives (hope for success and fear of failure), achievement orientations (win and goal orientation), SMI	An achievement-oriented motivational attitude which is also expressed phenotypically has a significant influence on the selection decisions of national coaches and is therefore an important talent criterion.
Zuber et al. (2016)	Longitudinal	3 years (TP1 – 2011; TP2 – 2012; TP3 – 2013)	Achievement motives (hope for success and fear of failure)	The importance of holistic approaches for predicting performance among promising soccer talents in the medium-term and thus provide valuable clues for their selection and promotion. However, none of the achievement-oriented, highly skilled, late-matured or late-matured, low skilled players reached the highest performance level.
Zuber et al. (2022)	Longitudinal	5 years (TP1 – 2017; TP2 – 2022)	Achievement motives (hope for success and fear of failure), achievement orientations (win and goal orientation), SDM, satisfaction	Players with a highly intrinsically achievement-oriented profile displayed a higher likelihood of reaching professional level. Motivated athletes showed specific behaviour that promotes the acquisition and development of motor characteristics

The studies tracked the players from 6 months (Verner-Filion and Gaudreau, 2023) to 15 years (Van Yperen, 2009), assessing achievement motives (hope for success and fear of failure) (Höner and Feichtinger, 2016; Zuber and Conzelmann, 2013; Zuber et al., 2014; Zuber et al., 2016; Zuber et al., 2022), self-determination and motivation (SMI, autonomous, controlled motivation) (Forsman et al., 2016; Hendry et al., 2019; Huijgen et al., 2014; Verner-Filion and Gaudreau, 2023; Zuber et al., 2014; Zuber et al., 2022), task and ego orientation (Figueiredo et al., 2009; Höner and Feichtinger, 2016; Huijgen et al., 2014), achievement goal orientation (competition, win and goal orientation) (Höner and Feichtinger, 2016; Zuber et al., 2014; Zuber et al., 2022), and self-confidence (Forsman et al., 2016; Höner and Feichtinger, 2016; Huijgen et al., 2014) as the most psychological factors analysed between 2009 and 2023.

*Achievement motives*. The AMS-S was used to measure the two motive components (i.e., hope for success and fear of failure) that provide information about the criteria that individuals use to define success and judge their level of ability. Five studies analyzed the relevance of achievement motives for future success. The results suggest that hope for success was related to future talent-like performance characteristics. Moreover, Höner and Feichtinger, 2016 found that hope for success was associated with future performance (*r*^2^ = 0.01; *p* < 0.05). However, Zuber et al. (2016) revealed a negative relationship between hope for success and fear of failure (*d* = −0.42).

*Self-determined motivation*. SDM was assessed by using different questionnaires, particularly: PSIS-R-5 (Forsman et al., 2016), PSIS-Youth (Huijgen et al., 2014), BRSQ2 (Hendry et al., 2019), and SMS (Verner-Filion and Gaudreau, 2023; Zuber et al., 2014; Zuber et al., 2022). Forsman et al. (2016) found a positive relationship between motivational skills and the future performance level of youth soccer players (*d* = 0.89; *p* < 0.01). Additionally, Zuber et al. (2014) indicated that talented soccer players with superior SDM were more likely to get selected to a higher performance level compared to players with lower SDM (*d* = 0.81, *p* < 0.01). In the same vein, Verner-Filion and Gaudreau (2023) found that high levels of need satisfaction were conducive to autonomous sport motivation (*β* = 0.392; *p* < 0.01), which was positively related to the likelihood of young players being selected in a competitive team. However, Hendry et al. (2019) found that SDM across age groups were not cohort specific, but rather indicative of trends within elite youth soccer (group interaction was not significant; *ηp^2^* = 0.06; *p* = 0.07). Moreover, Huijgen et al. (2014) did not find motivational skills to be significant “predictors” (*d* = 0.09), and these skills were negatively associated with future success. In sum, the researchers’ tendency to seek growth and embrace challenges to characterize the talent players need a methodological consensus.

*Task and ego orientation.* Three studies examined task and ego orientation using the TEOSQ (Figueiredo et al., 2009; Höner and Feichtinger, 2016; Huijgen et al., 2014). Figueiredo et al. (2009) did not relate both characteristics to the process of dropping out, persisting at the same competitive standard or moving up to a higher level of soccer players (task, *F* = 0.33; Ego, *F* = 0.05). Similar results were obtained by Huijgen et al. (2014), who did not find that task (*d* = 0.20) or ego orientation (*d* = 0.05) differentiated selected from deselected players. However, Höner and Feichtinger (2016) found that task orientation showed a small effect size (*F* = 0.36; *d* = 0.26, *p* < 0.05) to prognose talent players.

*Achievement orientation.* The use of SOQ distinguishes between three different achievement orientations (competition, win, and goal). These have a strong resemblance to the ego and task orientation scales (Duda, 1992). Höner and Feichtinger (2016) evidenced that competition (*F* = 6.20, *η_p_^2^* = 0.01; *p* < 0.05) and goal orientation (*F* = 4.25, *η_p_^2^* = 0.01; *p* < 0.05) revealed relevant associations with current and future soccer success. Albeit the description of the results was not explicit, Zuber et al. (2014) and Zuber et al. (2022) also stated that intrinsically achievement-oriented profile displayed a higher likelihood of reaching professional level.

*Self-confidence.* Self-confidence was assessed using the PSIS-R-5 (Forsman et al., 2016), VCS (Höner and Feichtinger, 2016), and PSIS-Youth (Huijgen et al., 2014). With regard to their prognostic value, Höner and Feichtinger (2016) demonstrated that self-optimization was more important than all three volitional deficits (*F* = 5.81, *η_p_^2^* = 0.01; *p* < 0.05). However, Huijgen et al. (2014) and Forsman et al. (2016) did not find any discriminatory factor of self-confidence to select or deselect talent player (*d* = 0.34; *d* = 0.27, *p* = 248; respectively).

*Other psychological characteristics*. A self-report measure of goal commitment and a questionnaire to assess coping was used by Van Yperen (2009), with the results suggesting a prognostic value of goal commitment (*η_p_^2^* = 0.14, *p* = 0.03) and engagement in problem-focused coping (*η_p_^2^* = 0.7, *p* = 0.04) for future success in soccer. Additionally, Höner and Feichtinger (2016) evidenced that anxiety components (i.e., concentration disruption, somatic anxiety) were not found to be prognostically significant (baseline, m = 6.80 ± 2.49; follow-up, m = 6.80 ± 2.46; *F* = 0.94). Moreover, Rongen et al. (2020) used simultaneously three different questionnaires (BMPN, KIDSCREEN-27, and AIMS) to assess the need of satisfaction, emotional well-being and athletic identity, which the results did not materialise the many concerns around negative psychosocial impacts of soccer academy involvement. However, heightened athletic identities remained a concern.

### Data syntheses

**Figure 2:**
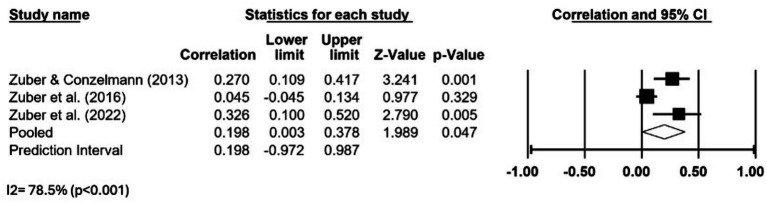
Forest plot describing the association between hope for success and talent identification. Values shown are effect sizes (Hedges’s g) with 95% confidence intervals (CI). The size of the plotted squares reflects the statistical weight of the study. White diamond: overall results.

Considering the reduced number of studies and their heterogeneity, only one meta-analysis could be performed. The results of the meta-analysis that aimed to examine the relationship between achievement motives and talent identification (assessed by hope for success) are described in Figure 2. The main analysis suggests that greater values of hope for success correlated positively with future talent-like performance, but the effect size was trivial (*g* = 0.198, 95% CI 0.003 to 0.378), and there was large impact of heterogeneity (*I*^2^ = 78.5%). The pooled estimate for achievement motive should be interpreted cautiously because it is based on a small number of studies, with substantial heterogeneity and limited independence among contributing samples. Accordingly, the meta-analytic result is presented as exploratory and as a summary of the current evidence base rather than as a definitive estimate of the relationship between psychological factors and future talent-like performance. In addition, it should be underlined that only three studies were available for this comparison, and all were conducted by the same lead author. Risk of reporting biases were not assessed, as no comparison reached the minimum required of 10 studies.

### Certainty of evidence

Given the impossibility to perform meta-analyses for most relevant parameters, the overall certainty of evidence surrounding the role of psychological factors on soccer talent should be considered very low. The single meta-analytical comparison that was possible to perform reflected the relationship between achievement motives and future talent-like performance. This analysis was based in only three studies from the same lead author, two of which judged at risk of bias and one at unclear risk of bias. Although an average positive effect size was found, with a 95% CI not crossing zero, this effect was trivial (<0.2) and accompanied by nearly 80% impact of heterogeneity. Therefore, certainty of evidence was deemed very low (Table 4).

**Table 4:** GRADE assessments for achievement motive and talent-like performance.

Outcome	*k (n)*	RoB	Indirectness	Risk of publication bias	Inconsistency	Imprecision	Certainty of evidence
Achievement motives and talent-like performance	3 (353)	Downgrade by 2 levels (high risk)	No downgrading (low by default)	—	Downgrade by 2 levels (*I*^2^ = 78.5%)	Downgrade by 1 level (<800 participants). Greater achievement motive favors future talent-like performance, but trivial effect size (<0.2)	⊕

Rules for judgment: (i) RoB: risk of bias in studies: downgrading by one level in the presence of moderate RoB and by two levels in the presence of high risk of bias. (ii) Indirectness: low by default given eligibility criteria. (iii) Risk of publication bias: not assessed (<10 studies per comparison). (iv) Inconsistency: downgraded by one level if *I*^2^ = 25–75% and by two levels if *I*^2^ > 75%. (vi) Imprecision: downgraded by one level if *n* < 800 (<400 per group; Guyatt et al., 2011) or effect direction unclear (95% CIs crossing zero), or by two levels if both occurred.

⊕: very low certainty of evidence. ⊕⊕: low certainty of evidence.

## Discussion

We systematically reviewed the psychological factors associated with talented soccer players across their developmental pathway, to critically appraise the psychological characteristics used in longitudinal research, and to summarize the current evidence to inform future studies and applied practice. Across 12 longitudinal studies and 3,748 players from recreational to elite levels, psychological characteristics were predominantly assessed via self-report questionnaires targeting achievement motives, motivational regulations, goal orientations, self-confidence, coping, and broader psychosocial adjustment (Van Yperen, 2009; Forsman et al., 2016; Höner and Feichtinger, 2016; Rongen et al., 2020; Verner-Filion and Gaudreau, 2023; Zuber and Conzelmann, 2013; Zuber et al., 2014, 2016, 2022). Despite the centrality of psychological factors in contemporary talent models (Bloom, 1985; Côté, 1999; Côté et al., 2007; Gledhill et al., 2017; Murr et al., 2018; Xiang et al., 2024), only one construct – achievement motives operationalized as hope for success – could be summarized meta-analytically, and its association with future talent-like performance was trivial, with substantial between-study heterogeneity and very low certainty of evidence (Zuber and Conzelmann, 2013; Zuber et al., 2014, 2016, 2022).

Taken together, the current literature suggests that psychological characteristics may contribute to talent development, but their isolated prognostic value for future performance appears modest, inconsistent, and highly sensitive to methodological choices (Murr et al., 2018; Ivarsson et al., 2020). This pattern is compatible with broader conceptual work that has classified psychological contributors to sports talent identification into psychological traits, motivation, and mental ability, highlighting personality, mental toughness, intrinsic motivation, self-esteem, and general mental ability as important domains to consider in TID models, but without demonstrating strong prospective effects on future performance (Xiang et al., 2024). It also aligns with multiple independent reviews (Gledhill et al., 2017; Murr et al., 2018; Ivarsson et al., 2020), which converge on the message that psychological factors show at best small effects, with considerable uncertainty, and cannot be used as standalone talent-selection tools.

### Achievement motives and motivational characteristics

Achievement motives, particularly hope for success, has been repeatedly proposed as a key marker of talented soccer players, as it reflects a tendency to pursue challenging goals, persist in the face of obstacles, and derive satisfaction from mastery and improvement (Höner and Feichtinger, 2016; Zuber and Conzelmann, 2013; Zuber et al., 2014, 2016, 2022; Duda, 1992). In this review, five studies examined achievement motives, and three from the same research group contributed to a meta-analysis that revealed a positive but trivial effect size for hope for success on future talent-like outcomes, with large heterogeneity and very low certainty (Zuber and Conzelmann, 2013; Zuber et al., 2014, 2016, 2022). At the individual-study level, higher hope for success was associated with subsequent performance in some cohorts (Höner and Feichtinger, 2016; Zuber et al., 2014), whereas the balance between hope for success and fear of failure (e.g., net hope) even showed negative relationships in others (Zuber et al., 2016), underscoring the instability of this construct as a standalone “predictor.” These findings suggest that while achievement-oriented profiles may align with expert coaches’ intuitions about “driven” players (Rees et al., 2016; Mann et al., 2017; Xiang et al., 2024), their isolated measurement through self-report scales is insufficient to yield robust prognostic information over multiple years of development.

Self-determined motivation and related constructs (e.g., autonomous vs. controlled regulation, motivational skills) were also investigated, but with mixed results (Forsman et al., 2016; Huijgen et al., 2014; Hendry et al., 2019; Verner-Filion and Gaudreau, 2023; Zuber et al., 2014, 2022). Some studies reported that higher motivational skills or more autonomous motivational profiles were associated with later selection to higher competitive levels, consistent with self-determination theory and prior reviews highlighting the importance of intrinsic motivation in talent development (Forsman et al., 2016; Zuber et al., 2014; Verner-Filion and Gaudreau, 2023; Gledhill et al., 2017; Xiang et al., 2024). However, other longitudinal cohorts did not find motivational characteristics to discriminate between selected and deselected players, and in at least one study motivational scores were negatively associated with future success (Hendry et al., 2019; Huijgen et al., 2014). This pattern reinforces previous calls for methodological standardization in psychological talent research, as inconsistent operationalization of motivation, different time horizons, and varying definitions of “success” make it difficult to aggregate or compare findings across studies (Murr et al., 2018; Ivarsson et al., 2020; Afonso et al., 2024).

### Goal orientations, volitional competencies, and other psychological factors

Task and ego orientations, as assessed by the TEOSQ, have been discussed in youth sport as indicators of adaptive versus maladaptive motivational climates (Duda, 1992; Roberts et al., 1998). In our review, task and ego orientation generally failed to correlate with future performance, dropout, or transitions between competitive levels, with only one study identifying a small prognostic effect of task orientation (Figueiredo et al., 2009; Höner and Feichtinger, 2016; Huijgen et al., 2014). Similarly, achievement orientations derived from the SOQ (competition, win, and goal orientation) were associated with current and future success in some samples, especially when embedded in broader achievement-oriented profiles, but these results were not consistently replicated across studies or analytic approaches (Höner and Feichtinger, 2016; Zuber et al., 2014, 2022).

Volitional competencies and self-confidence were also examined as potential talent markers (Forsman et al., 2016; Höner and Feichtinger, 2016; Huijgen et al., 2014). Evidence suggested that self-optimization might be more relevant than volitional deficits for future performance in one cohort (Höner and Feichtinger, 2016), whereas other longitudinal studies did not detect discriminatory effects of self-confidence to distinguish selected from deselected players (Huijgen et al., 2014; Forsman et al., 2016). Additional psychological constructs such as goal commitment, coping styles, basic psychological need satisfaction, emotional well-being, and athletic identity showed heterogeneous findings: goal commitment and problem-focused coping displayed some prognostic value for career success (Van Yperen, 2009), whereas concerns about negative psychosocial effects of academy involvement were not supported in one study, despite consistently high and exclusive athletic identities (Rongen et al., 2020). Collectively, these results indicate that a broad range of psychological characteristics may be functionally involved in talent development, but their predictive contribution appears context-dependent and modest when considered in isolation from technical, tactical, physical, and environmental variables (Sarmento et al., 2018; Mann et al., 2017; Xiang et al., 2024).

### Methodological considerations and certainty of evidence

From a methodological standpoint, the current body of longitudinal evidence on psychological correlates of soccer talent exhibit several challenges that limit firm conclusions (Murr et al., 2018; Ivarsson et al., 2020). First, all studies used self-report questionnaires, often with different instruments targeting conceptually overlapping constructs, which introduces measurement heterogeneity and complicates direct comparisons or pooled analyses (Forsman et al., 2016; Höner and Feichtinger, 2016; Huijgen et al., 2014; Hendry et al., 2019; Verner-Filion and Gaudreau, 2023; Zuber et al., 2014, 2016, 2022). Second, risk of bias was high across the board: 10 of the 12 studies had at least one to three domains rated at high risk, mainly due to confounding and incomplete outcome data, and none of the investigations had a pre-registered protocol (Park et al., 2011; Ivarsson et al., 2020; Afonso et al., 2024). Third, important confounders such as biological maturation, training history, position, and changes in coaching or club environments were rarely monitored systematically, despite their known relevance for performance trajectories in youth soccer (Barreiros et al., 2013; Berry et al., 2008; Coelho e Silva et al., 2010; Bidaurrazaga-Letona et al., 2019; Sarmento et al., 2018).

Certainty of evidence for the only meta-analytic comparison – achievement motives and talent-like performance – was rated very low, after downgrades for high risk of bias, substantial inconsistency, and imprecision arising from fewer than 800 participants and wide confidence intervals (Guyatt et al., 2011; Schünemann et al., 2020a, 2020b; Afonso et al., 2024). For all other constructs, the impossibility of conducting meta-analyses due to insufficient or heterogeneous data meant that the certainty of evidence was also judged to be very low (Guyatt et al., 2011; Schünemann et al., 2020a, 2020b).

These findings mirror the broader talent development literature, where small samples, unstandardized outcome definitions, and short follow-up periods have repeatedly undermined attempts to identify stable and generalizable psychological correlates of elite performance (Bloom, 1985; Gledhill et al., 2017; Murr et al., 2018; Ivarsson et al., 2020; Xiang et al., 2024). Conceptual classifications of psychological factors in talent identification, while valuable for framing practice and scale development, must be complemented by rigorous longitudinal evidence if psychological constructs are to inform selection decisions in a robust and defensible way (Xiang et al., 2024).

### Limitations

This review has several limitations that should be acknowledged when interpreting the findings. The search strategy was deliberately designed to prioritize studies situated within the football talent identification and development framework, ensuring conceptual specificity. This approach may have reduced sensitivity for psychological studies that did not explicitly use talent or future performance terminology, and this trade-off should be considered when interpreting the breadth of the evidence base. At the evidence level, only 12 longitudinal studies met the inclusion criteria from an initial pool of 4,015 records, indicating that prospective research on psychological predictors of soccer talent remains scarce relative to the volume of cross-sectional and multi-factorial work. The concepts of “talent” and “future performance” were operationalized heterogeneously across studies, including selection/deselection, progression to higher competitive levels, contract attainment, and coach-rated performance. This variability complicates direct comparison across studies and likely contributed to the substantial heterogeneity observed in the meta-analysis. Moreover, the included studies were characterized by heterogeneous samples in terms of age, sex, and competitive level, with a predominance of male players and relatively few female participants, which restricts generalization to women’s soccer. Moreover, psychological constructs were operationalized using different instruments and scales, often with limited overlap across studies, which precluded comprehensive quantitative synthesis beyond achievement motives. Finally, the provided studies were conducted over a maximum of 2 years, which is a rather short prognostic period considering the duration of the talent development process. Thus, future analyses should extend those periods to examine the midterm or even long-term prognostic value of psychological characteristics.

At the review process level, the meta-analysis was restricted to outcomes with at least three contributing studies, and this threshold was only reached for achievement motives, all from the same lead author. This concentration of evidence raises concerns about the robustness and replicability of the pooled effect, as it may reflect specific methodological choices, sample characteristics, or analytical strategies rather than a generalizable relationship. Although rigorous eligibility criteria based on the P.E.C.O.S. framework and PRISMA 2020 guidelines were applied, and RoBANS and GRADE were used to formally appraise risk of bias and certainty of evidence, the overall strength of the conclusions remains constrained by the underlying primary literature rather than by the review methods themselves.

### Practical implications

Despite very low certainty of evidence, some provisional implications may be derived for researchers and practitioners involved in talent detection, identification, and development in soccer. First, psychological characteristics should not be used as standalone selection or deselection criteria, particularly when based on single time-point self-report measures; instead, they should be integrated into holistic, multi-dimensional assessments that combine technical, tactical, physical, and contextual information. Second, constructs such as achievement motives, self-determined motivation, goal commitment, and problem-focused coping may be more appropriately viewed as developmental targets for psychological skills training and environmental design, rather than as fixed traits that neatly distinguish future elite from non-elite players.

From a research perspective, future studies should prioritize longitudinal, multi-cohort designs that extend beyond two-year horizons, to better capture the dynamic interplay between psychological characteristics and evolving performance levels over mid- and long-term developmental periods. The consistent use of validated instruments, harmonized outcome definitions (e.g., talent-like performance, contract attainment, league level), and standardized reporting of confounders such as maturation, training load, and coaching context would markedly improve comparability and the feasibility of meta-analytic syntheses. Pre-registration of protocols, transparent handling of missing data, and systematic reporting of all measured outcomes are also essential to mitigate selective reporting and enhance the credibility of findings.

## Conclusion

This systematic review with meta-analysis synthesized the longitudinal evidence on psychological characteristics associated with talented soccer players, focusing on studies that tracked players over time and contrasted those classified as talented with non-talented peers. Across available investigations, psychological factors such as achievement motives, self-determined motivation, goal orientations, volitional competencies, and coping processes showed occasional associations with future performance, but these effects were generally small, inconsistent, and derived from studies at high risk of bias. The only construct amenable to meta-analysis, achievement motives indexed by hope for success, demonstrated a trivial positive association with future talent-like performance, with substantial heterogeneity and very low certainty of evidence. Overall, the current literature does not support the use of any single psychological characteristic as a robust prognostic marker of soccer talent, reinforcing the view that talent development is a multifactorial, dynamic process emerging from the interaction of psychological, technical, tactical, physical, and environmental factors. Moreover, other psychological characteristics such as mental toughness, grit, and resilience may play a functional role in talent development, but their isolated prognostic value for future performance appears small, inconsistent, and highly sensitive to methodological choices. Strengthening this field will require coordinated efforts to implement rigorous, pre-registered longitudinal designs rather than solely cross-sectional designs, harmonized measurement strategies, and ecologically valid outcomes that reflect the complex realities of soccer talent pathways. Future longitudinal studies should prioritize more diverse samples, particularly female cohorts, to strengthen the external validity of the evidence base.

## Data Availability

The original contributions presented in the study are included in the article/Supplementary material, further inquiries can be directed to the corresponding author.
