# Supplementary material for: The psychological characteristics behind talented soccer players: a systematic review with meta-analysis
Source: Front Psychol. 2026 May 6;17:1821294. doi: 10.3389/fpsyg.2026.1821294 (PMC13188071; doi:10.3389/fpsyg.2026.1821294)
Supplement: Supplementary file 2 [file Supplementary_file_2.docx]

***Supplementary Table 2*.** Full search strategies for each database.

| **Database** | **Search strategy** |
| --- | --- |
| PubMed | ((Soccer [Title/Abstract] OR Football*[Title/Abstract]) AND *(Talent** [Title/Abstract])) |
| Scopus | ((TITLE-ABS (Soccer OR Football*) AND (Talent*)) |
| Web of  Science | ALL FIELDS: (Soccer OR Football* AND *Talent*)* |
